# Methylome-wide association analyses of lipids and modifying effects of behavioral factors in diverse race and ethnicity participants

**DOI:** 10.1186/s13148-025-01859-3

**Published:** 2025-04-02

**Authors:** Yao Hu, Jeff Haessler, Jessica I. Lundin, Burcu F. Darst, Eric A. Whitsel, Megan Grove, Weihua Guan, Rui Xia, Mindy Szeto, Laura M. Raffield, Scott Ratliff, Yuxuan Wang, Xuzhi Wang, Alison E. Fohner, Megan T. Lynch, Yesha M. Patel, S. Lani Park, Huichun Xu, Braxton D. Mitchell, Joshua C. Bis, Nona Sotoodehnia, Jennifer A. Brody, Bruce M. Psaty, Gina M. Peloso, Michael Y. Tsai, Stephen S. Rich, Jerome I. Rotter, Jennifer A. Smith, Sharon L. R. Kardia, Alex P. Reiner, Leslie Lange, Myriam Fornage, James S. Pankow, Mariaelisa Graff, Kari E. North, Charles Kooperberg, Ulrike Peters

**Affiliations:** 1 Division of Public Health Sciences, Fred Hutchinson Cancer Center, Seattle, WA USA; 2 Department of Epidemiology, University of North Carolina, Chapel Hill, NC USA; 3 School of Public Health, Human Genetics Center, University of Texas Health Sciences Center at Houston, Houston, TX USA; 4 Division of Biostatistics and Health Data Science, School of Public Health, University of Minnesota, Minneapolis, MN USA; 5 McGovern Medical School, The Brown Foundation Institute of Molecular Medicine, The University of Texas Health Science Center at Houston, Houston, TX USA; 6 Department of Epidemiology, Colorado School of Public Health, Aurora, CO USA; 7 Department of Genetics, University of North Carolina, Chapel Hill, NC USA; 8 Department of Epidemiology, School of Public Health, University of Michigan, Ann Arbor, MI USA; 9 Department of Biostatistics, Boston University School of Public Health, Boston, MA USA; 10 Cardiovascular Health Research Unit, Department of Medicine, University of Washington, Seattle, WA USA; 11 Department of Medicine, University of Maryland School of Medicine, Baltimore, MD USA; 12 Center for Immuno-Oncology, Center for Cancer Research, National Cancer Institute, National Institutes of Health, Bethesda, MD USA; 13 Department of Preventive Medicine, Keck School of Medicine, University of Southern California, Los Angeles, CA USA; 14 Population Sciences in the Pacific Program, University of Hawaii Cancer Center, Honolulu, HI USA; 15 Department of Laboratory Medicine and Pathology, University of Minnesota, Minneapolis, MN USA; 16 Center for Public Health Genomics, University of Virginia, Charlottesville, VA USA; 17 Department of Pediatrics, The Institute for Translational Genomics and Population Sciences, The Lundquist Institute for Biomedical Innovation at Harbor-UCLA Medical Center, Torrance, CA USA; 18 Department of Epidemiology, University of Washington, Seattle, WA USA; 19 Human Genome Sequencing Center, Baylor College of Medicine, Houston, TX USA; 20 Division of Epidemiology and Community Health, School of Public Health, University of Minnesota, Minneapolis, MN USA

## Abstract

Circulating lipid concentrations are clinically associated with cardiometabolic diseases. The phenotypic variance explained by identified genetic variants remains limited, highlighting the importance of searching for additional factors beyond genetic sequence variants. DNA methylation has been linked to lipid concentrations in previous studies, although most of the studies harbored moderate sample sizes and exhibited underrepresentation of non-European ancestry populations. In addition, knowledge of nongenetic factors on lipid profiles is extremely limited. In the Population Architecture Using Genomics and Epidemiology (PAGE) Study, we performed methylome-wide association analysis on 9,561 participants from diverse race and ethnicity backgrounds for HDL-c, LDL-c, TC, and TG levels, and also tested interactions between smoking or alcohol intake and methylation in their association with lipid levels. We identified novel CpG sites at 16 loci (*P* < 1.18E-7) with successful replication on 3,215 participants. One additional novel locus was identified in the self-reported White participants (*P* = 4.66E-8). Although no additional CpG sites were identified in the genome-wide interaction analysis, 13 reported CpG sites showed significant heterogeneous association across smoking or alcohol intake strata. By mapping novel and reported CpG sites to genes, we identified enriched pathways directly linked to lipid metabolism as well as ones spanning various biological functions. These findings provide new insights into the regulation of lipid concentrations.

**Supplementary Information:**

The online version contains supplementary material available at 10.1186/s13148-025-01859-3.

## Introduction

Circulating lipid concentrations are clinically associated with cardiometabolic diseases [1, 2]. Genome-wide association studies (GWAS) and whole-exome sequencing (WES)/whole genome sequencing (WGS) have identified thousands of genetic loci associated with high-density lipoprotein cholesterol (HDL-c), low-density lipoprotein cholesterol (LDL-c), total cholesterol (TC), and triglycerides (TG) [3–5]. Similar to most complex polygenic traits, however, the phenotypic variances explained by these identified genetic loci remain limited and the underlying mechanisms remain to be fully understood [5].

More recently, blood DNA methylation has been linked to circulating lipid concentrations in several studies [6–12]. Over 900 unique CpG sites have been associated with HDL-c, LDL-c, TC, and TG levels, highlighting the importance of looking for additional factors contributing to lipid variations beyond genetic sequence variants. All these published studies focused solely on European populations, except one study [12]. African Americans and Hispanic/Latino participants together with European participants were included in an effort from the Cohort for Heart and Aging Research in Genomic Epidemiology (CHARGE) Consortium, uncovering both population-agnostic and population-specific novel findings and relatively low correlation of effect estimates of LDL-associated CpG sites across population groups [12]. These results emphasize the need to improve diversity and inclusion in epigenetic studies.

Previous studies have reported the modifying effects of nongenetic factors on lipid-gene associations, including smoking and alcohol intake, leading to identifications of additional novel loci for lipids [13, 14]. Recent studies also demonstrated the effect of smoking and alcohol intake on DNA methylation changes [15–17]. However, no study has explored potential modifying effects of smoking or alcohol intake on associations between lipid levels and DNA methylation variation using interaction analysis.

Here, we first present methylome-wide association analyses for HDL-c, LDL-c, TC, and TG in over 12,000 self-reported White, African American, Hispanic/Latino, Asian American, and Native American participants from eight studies. We then performed interaction analyses taking into account smoking and alcohol intake. We aim to identify novel CpG sites associated with HDL-c, LDL-c, TC, and TG levels in a diverse race and ethnicity setting and to explore the potential effect of smoking and alcohol intake on the association between CpG sites and lipid levels.

## Methods

### Study populations

A total of eight studies comprising over 12,000 participants were included in the analysis. Three studies, the Atherosclerosis Risk in Communities (ARIC) study [18, 19], the Women’s Health Initiative (WHI) [20], and the Jackson Heart Study (JHS) [21], were included in the discovery stage. An additional five studies, the Amish study [22, 23], the Cardiovascular Health Study (CHS) [24], the Genetic Epidemiology Network of Arteriopathy (GENOA) [25], the Multi-ethnic Cohort (MEC) study [26], and the Multi-Ethnic Study of Atherosclerosis (MESA) study [27], were included in the replication stage. Self-reported White, African American, and Hispanic/Latino participants formed the three largest population groups. Smaller numbers of Asian American and Native American participants were also represented in our analysis. Each participating study is detailed in the Supplemental Methods. All studies obtained written informed consent from participants and were approved by local institutional review boards and ethics committees.

### Lipid measurements

HDL-c, TC, and TG levels (mg/dL) in fasting blood were measured while LDL-c levels were calculated using the Friedewald equation. LDL-c levels were not calculated if the corresponding TG levels were greater than 400 mg/dL. Lipid levels were further adjusted for medication use by adding a constant based on previous publications (Supplemental Table 1) [28]. If multiple medications were used, only the largest constant was applied. Participants who were pregnant at blood draw or who had fasted less than eight hours prior to blood draw were excluded from the analysis. TG levels after adjustment for medication were natural log transformed.

**Table 1:** Basic characteristics of participating studies^a^

Study	Chip	N (% female)	Self-reported race and ethnicity	Age (years)	HDL-c (mg/dL)	LDL-c (mg/dL)	TC (mg/dL)	TG (mg/dL) ^b^
*Discovery studies*								
ARIC	450 k	1090 (58.5)	White	60.4 (5.4)	52.0 (18.2)	130.5 (36.8)	210.6 (38.6)	4.8 (0.5)
		2274 (62.7)	African American	57.4 (5.9)	53.1 (17.8)	134.5 (40.5)	211.1 (42.4)	4.6 (0.5)
WHI	450 k	2674 (100)	White	69.9 (9.5)	56.0 (14.8)	144.4 (36.2)	230.5 (39.5)	4.9 (0.5)
		1451 (100)	African American	65.2 (8.8)	56.8 (14.8)	147.5 (43.6)	227.8 (46.6)	4.7 (0.4)
		712 (100)	Hispanic/Latino	61.5 (6.6)	52.7 (14.1)	140.6 (38.0)	226.4 (42.1)	5.0 (0.5)
		125 (100)	Asian American	65.4 (7.1)	60.1 (14.2)	125.0 (31.8)	219.7 (35.5)	5.0 (0.5)
		48 (100)	Native American	64.0 (7.9)	54.2 (15.4)	134.0 (36.3)	222.5 (41.5)	5.0 (0.5)
JHS	EPIC	1187 (61.7)	African American	56.9 (11.6)	51.3 (14.7)	134.9 (34.6)	208.0 (43.0)	4.6 (0.5)
*Replication studies*								
Amish	450 k	346 (53.0)	White	57.1 (13.6)	56.1 (14.6)	145.8 (43.5)	219.5 (48.0)	4.3 (0.5)
CHS	450 k	419 (60.1)	White	75.0 (4.9)	52.4 (14.4)	121.6 (32.8)	204.4 (38.5)	4.9 (0.5)
		324 (70.8)	African American	73.1 (5.5)	57.4 (15.5)	122.2 (35.3)	202.1 (39.1)	4.6 (0.5)
GENOA	EPIC	943 (71.6)	African American	57.5 (10.3)	54.8 (16.8)	123.8 (42.0)	206.9 (45.7)	4.9 (0.4)
MEC	EPIC	67 (67.2)	African American	65.0 (7.1)	46.8 (19.0)	141.2 (41.7)	208.7 (45.6)	4.6 (0.5)
		91 (41.8)	Hispanic/Latino	67.0 (6.6)	41.7 (14.0)	130.9 (37.6)	205.7 (44.0)	5.0 (0.5)
		34 (23.5)	Asian American	63.0 (7.5)	43.2 (18.7)	112.3 (48.2)	184.0 (50.3)	4.8 (0.6)
		57 (50.9)	American Indian	64.4 (6.3)	40.1 (13.4)	126.8 (39.8)	188.9 (42.3)	4.6 (0.5)
MESA	EPIC	395 (50.0)	White	61.0 (9.9)	51.2 (14.7)	128.1 (30.5)	208.0 (36.9)	4.8 (0.5)
		181 (58.0)	African American	61.0 (9.6)	51.6 (14.8)	122.7 (37.5)	195.1 (42.5)	4.5 (0.5)
		287 (55.0)	Hispanic/Latino	58.6 (9.4)	47.6 (12.2)	125.2 (34.9)	202.8 (39.6)	4.9 (0.5)
		71 (46.0)	Asian American	60.8 (10.1)	48.4 (11.8)	129.1 (11.8)	211.4 (41.9)	5.0 (0.6)

HDL-c, high-density lipoprotein; LDL-c, low-density lipoprotein; TC, total cholesterol; TG, triglycerides

^a^Values are shown in mean (SD) if not specified

^b^TG values are natural log transformed

### Smoking and alcohol intake measurements

To explore the potential modifying effects of behavioral factors on associations between DNA methylation and lipid levels, we collected information on smoking and alcohol intake in each study at the time of blood draw (Supplemental Table 2), for exploration of potential interactions. According to smoking behavior, participants were divided into three groups: nonsmokers, past smokers, and current smokers. According to alcohol intake and sex, participants were divided into four groups: non-drinkers, light drinkers (≤ 3 servings of alcohol intake per week), moderate drinkers (3 < servings of alcohol intake per week ≤ 7 and 3 < servings of alcohol intake per week ≤ 14 for female and male, respectively), and heavy drinkers (> 7 and > 14 servings of alcohol intake per week for female and male, respectively). Amish and MEC studies were excluded from the analysis due to extremely limited sample sizes (*n* < 50) in heavy drinkers and/or current smoker groups.

**Table 2:** Novel CpG sites identified in the population-combined and population-specific meta-analysis^a^

Marker	Chr:pos	Trait	Gene	Population-combined meta-analysis (*N* = 9,561)		Meta-analysis in White participants (*N* = 3,764)		Meta-analysis in African American participants (*N* = 4,912)		Meta-analysis in Hispanic/Latino participants (*N* = 712)	
				Beta (SE)	*P*	Beta (SE)	*P*	Beta (SE)	*P*	Beta (SE)	*P*
*Novel CpG sites identified in the population-combined meta-analysis*											
cg17438457	1:53,094,893	HDL	–	−3.02 (0.43)	2.13E-12	−3.35 (0.68)	4.42E-7	−2.19 (0.71)	2.13E-3	−3.56 (1.50)	1.75E-2
cg00522451	2:113,464,048	HDL	–	1.68 (0.30)	2.29E-8	1.81 (0.55)	5.83E-4	1.75 (0.41)	1.99E-5	0.31 (1.12)	7.79E-1
cg18194850	3:67,699,305	HDL	*SUCLG2*	−1.60 (0.26)	1.10E-9	−2.22 (0.46)	9.00E-7	−1.19 (0.37)	1.28E-3	−1.08 (1.03)	2.95E-1
cg01671681	3:155,421,735	HDL	*PLCH1*	1.43 (0.24)	2.19E-9	1.50 (0.38)	4.26E-5	1.25 (0.37)	6.28E-4	2.77 (0.97)	4.26E-3
cg02387843	4:9,892,887	HDL	*SLC2A9*	2.10 (0.30)	4.54E-12	1.73 (0.51)	3.48E-4	2.39 (0.45)	8.80E-8	2.25 (1.19)	5.85E-2
cg16905822	10:924,646	HDL	*LARP4B*	−2.24 (0.40)	2.40E-8	−2.28 (0.65)	3.47E-4	−2.38 (0.61)	9.57E-5	−0.81 (1.52)	5.97E-1
cg23669118	16:1,538,347	HDL	*C16orf38*	−1.76 (0.29)	1.37E-9	−1.71 (0.50)	5.49E-4	−1.67 (0.41)	5.20E-5	−2.57 (1.14)	2.34E-2
cg24403644	20:42,574,624	HDL	*TOX2*	−2.55 (0.43)	3.30E-9	−2.32 (0.72)	1.09E-3	−2.91 (0.63)	4.50E-6	−1.04 (1.57)	5.07E-1
cg03607951	1:79,085,586	LDL	*IFI44L*	0.76 (0.12)	3.47E-10	0.75 (0.23)	1.14E-3	0.78 (0.16)	8.16E-7	0.13 (0.63)	8.34E-1
cg02387843	4:9,892,887	TG	*SLC2A9*	−1.83 (0.32)	1.24E-8	−1.45 (0.43)	7.79E-4	−2.07 (0.41)	5.01E-7	−2.25 (1.25)	7.20E-2
cg00960906	5:31,769,846	TG	*-*	−1.63 (0.30)	7.87E-8	−2.35 (0.44)	7.48E-8	−1.02 (0.37)	5.87E-3	−1.44 (1.19)	2.25E-1
cg07398791	5:118,676,053	TG	*TNFAIP8*	−1.81 (0.28)	1.13E-10	−1.67 (0.37)	8.26E-6	−2.07 (0.36)	1.05E-8	0.09 (1.04)	9.28E-1
cg18722504	5:139,712,966	TG	*HBEGF*	−2.39 (0.43)	2.97E-8	−2.97 (0.59)	4.11E-7	−1.70 (0.55)	2.02E-3	−3.40 (1.64)	3.85E-2
cg14761417	7:130,636,860	TG	*FLJ43663*	−2.38 (0.33)	1.40E-12	−2.13 (0.47)	7.25E-6	−2.20 (0.41)	1.06E-7	−5.06 (1.30)	9.79E-5
cg04927537	17:76,976,091	TG	*LGALS3BP*	1.11 (0.18)	2.42E-9	1.22 (0.28)	1.53E-5	1.11 (0.22)	2.53E-7	−0.18 (0.73)	8.01E-1
cg24673765	19:36,247,869	TG	*HSPB6*	1.89 (0.35)	1.07E-7	1.47 (0.53)	5.64E-3	2.11 (0.42)	4.13E-7	1.30 (1.40)	3.55E-1
cg24403644	20:42,574,624	TG	*TOX2*	2.84 (0.46)	4.87E-10	2.10 (0.62)	6.81E-4	3.43 (0.59)	5.05E-9	2.83 (1.65)	8.62E-2
cg04945608	20:43,118,723	TG	*TTPAL*	−2.34 (0.36)	1.10E-10	−2.21 (0.52)	2.03E-5	−2.22 (0.44)	4.46E-7	−3.65 (1.44)	1.11E-2
*Novel CpG site identified in the EA-specific meta-analysis*											
cg03584506	11: 39,689,828	TG	-	−0.99 (0.22)	4.29E-6	−1.94 (0.36)	4.66E-8	−0.55 (0.24)	2.24E-2	−1.05 (0.90)	2.44E-1

HDL, high-density lipoprotein; LDL, low-density lipoprotein; TC, total cholesterol; TG, triglycerides

^a^Association results presented in this table are derived from the discovery stage. Detailed results in the replication stage are presented in Supplemental Table 4–6

### DNA methylation measurement, quality control, and normalization

DNA methylation was quantified in each participating study independently. Levels were measured from peripheral blood leukocytes isolated from whole blood. The Illumina Infinium HumanMethylation450 BeadChip was used in the Amish, ARIC, CHS, and WHI studies, while the Illumina Infinium MethylationEPIC BeadChip was used in the GENOA, JHS, MEC, and MESA studies. Either the Beta MIxture Quantile dilation (BMIQ) [29], the normal-exponential out-of-band (Noob) [30], or the subset quantile normalization [31] approach was used to perform preprocessing and normalization in each study. A beta value was calculated for each CpG site representing the percentage of methylation at that site. Houseman’s method was implemented to estimate white blood cell proportions [32]. Any single value with a detection *p* value > 0.01 was set to missing. Probes with missing data in greater than 5% of samples per study were excluded. Samples with greater than 5% of missing probes were also excluded. To avoid spurious signals in DNA methylation data, we excluded CpG sites that co-hybridize to alternate genomic sequences or overlapped with genomic variations.

### Methylome-wide association analysis and meta-analysis

Methylome-wide association analysis was performed in each study stratified by self-reported race and ethnicity groups, followed by population-combined and population-specific meta-analysis. In the study- and population-specific methylome-wide association analysis, we first regressed the four lipid traits on age, sex, and study-specific covariates (if applicable) and estimated the residual values. In the next step, the inverse-normalized residual values were used as the outcome for testing association with each CpG site using linear mixed models. White blood cell proportions (CD8T, CD4T, NK, BCELL, MONO, and GRAN values estimated using the Houseman’s method [30]), the first 10 principal components (PCs, except in Amish, where a mixed model controlling for the fixed effects of covariates, and the random effect of a genetic relationship matrix derived from the complete Amish pedigree structure was used [https://mmap.github.io/]), row, and column were adjusted as fixed-effect covariates and chip was adjusted as random-effect covariates. Summary statistics from each study and ancestral group were combined through fixed-effect inverse-variance weighted meta-analysis using METAL [33]. Population-specific meta-analysis was performed in African American, White, and Hispanic/Latino participants, which are the three largest population groups in our analysis. To control for bias and inflation, BACON [34] adjustment was implemented for the meta-analysis results generated by METAL. CpG sites with *P* < 1.18E-7 were considered as genome-wide significant. These analyses focused only on CpG sites that are available on the HumanMethylation450 BeadChip to maximize sample sizes. Due to the limited sample sizes of studies using the MethylationEPIC BeadChip, we summarized association results of CpG sites on the MethylationEPIC BeadChip by combining all available studies in our analysis.

### Methylome-wide interaction analysis with smoking and alcohol intake

We performed methylome-wide interaction analysis in all eight participating studies except Amish and MEC due to limited sample sizes. Methylome-wide interaction analysis (which has two degrees of freedom for the three-level smoking variable and three degrees of freedom for the four-level alcohol variable) was performed in each study stratified by population groups, followed by population-combined and population-specific meta-analysis in the discovery stage and in the replication stage. In the methylome-wide interaction analysis, we used the inverse-normalized residual values as the outcome and adjusted for the same set of covariates as in the methylome-wide association analysis. To combine study- and population-specific results, we estimated a test statistic based on coefficients and variance–covariance matrix from each study and population group. This test statistic followed a χ^2^ distribution and was used to estimate *P* values. Genomic control was applied to the *P* values to account for inflation. CpG sites with *P* < 1.18E-7 were considered as genome-wide significant.

### Stratified association analysis across smoking and alcohol intake strata

We then focused our efforts on better characterizing previously reported CpG sites and novel CpG sites identified in our methylome-wide association analysis in terms of their potential interactions with smoking and alcohol intake. Association analysis between each CpG site and each lipid level was performed in each smoking (non, past, and current) and alcohol intake stratum (none, light, moderate, and heavy) with the same adjustment implemented in the methylome-wide association analysis. *P* values for heterogeneity were estimated across different strata and the significant threshold was set as *P* < 4.01E-5 (0.05/1246 CpG sites tested). Stratified analyses were performed only in the three discovery studies (ARIC, WHI, and JHS, Supplemental Table 2).

### Pathway enrichment analysis

Gene ontology (GO) for biological processes (BP) was used to analyze the differentially methylated genes for functional enrichment using the R package “clusterProfiler” [35, 36]. We combined all previously reported CpG sites with the novel ones we identified in our meta-analysis for the pathway enrichment analysis. The annotations of these CpG sites to the corresponding genes were performed using the R package “missMethyl” [37]. Associations that were *P* < 0.05 after FDR adjustment were considered statistically significant.

## Results

Participating studies were divided into discovery (ARIC, WHI, and JHS) and replication stages (Amish, CHS, GENOA, MEC, and MESA). Mean ages across studies ranged from 56.9 (JHS African American) to 75.0 (CHS white, Table 1). Smoking and alcohol intake status for participants in each study are summarized in Supplemental Table 2.

### Novel CpG sites associated with lipids

In the discovery stage, a total of 9,561 participants from ARIC, WHI, and JHS were analyzed using both population-combined and population-specific approaches. In the African American, White, and Hispanic/Latino populations, the sample sizes were 4,912, 3,764, and 712, respectively. Novel CpG sites identified in the discovery stage (*P* < 1.18E-7 and located > 500 kb away from any reported CpG sites) were carried forward for replication in 3,215 participants from the Amish, CHS, GENOA, MEC, and MESA studies. For this part, we focused on CpG sites available on the HumanMethylation450 BeadChip to maximize sample sizes. Across all analyses, lambda values ranged from 0.997 to 1.258 after BACON adjustment, indicating limited amount of inflation (Supplemental Table 3).

In the population-combined analysis, a total of 348 CpG sites mapped to 303 loci (based on the genomic locations of CpG sites and each locus is > 500 kb away from each other) were significantly associated with at least one of the four lipid traits in the discovery stage (Supplemental Table 4). More than half of these CpG sites also displayed consistent directions of association in the replication stage (190 CpG sites mapped to 175 loci, Supplemental Table 4). However, only eight, one, and nine loci were successfully replicated in the replication stage for HDL-c, LDL-c, and TG, respectively (*P* < 0.05/number of significant loci for each lipid trait, Table 2). In the African American- and White-specific meta-analysis, 12 and four additional novel CpG sites were identified, respectively (Supplemental Table 5), and one of them was successfully replicated for association with TG in self-reported White participants (*P* < 3.13E-3, 0.05/16 significant loci in African American- and White-specific meta-analysis, Table 2). The limited sample sizes in the replication stage compared to the discovery stage (3,215 and 9,516 participants, respectively) could have an impact on the statistical power and the failure of replication. In summary, we identified and replicated 17 novel CpG sites in the multi-population meta-analysis.

To maximize sample sizes for CpG sites only available on the MethylationEPIC BeadChip, we performed meta-analysis including all studies and summarized 96 top CpG sites that have not been previously reported in Supplemental Table 6 (*P* < 1.18E-7).

### Replication of previously reported CpG sites

In order to assess the generalizability of previously identified CpG sites from the literature to our diverse populations in PAGE, we evaluated previously reported CpG sites in our population-combined and population-specific meta-analysis from the discovery stage (Supplemental Tables 7–10). In the population-combined analysis, 20.4% (383 out of 1,876), 21.0% (69 out of 328), 7.8% (14 out of 180), and 19.4% (351 out of 1,824) reached genome-wide significance for association with HDL-c, LDL-c, TC, and TG, respectively (*P* < 1.18E-7 and with consistent association directions of the reported trait, Supplemental Table 7). The percentages increased to 57.1%, 59.5%, 60.6%, and 57.6% for HDL-c, LDL-c, TC, and TG, respectively, when setting the *P* value cutoff to 0.05 (with consistent association directions of the reported trait, Supplemental Table 7). In the White- and African American-specific meta-analysis, the percentages of reported CpG sites showing genome-wide significance dropped to a maximum of 12.8% while the percentages of reported CpG sites with *P* < 0.05 stayed close to 50% (Supplemental Tables 8–9). In the Hispanic/Latino-specific meta-analysis with a considerably smaller sample size, the percentages of reported CpG sites showing genome-wide significance were all below 1% and roughly 15% reported CpG sites showed *P* < 0.05 (Supplemental Table 10). It is worth mentioning that the vast majority of previously reported loci (85.4%) are reported by the recent multi-ethnic meta-analysis [12] and there are overlapping samples (60.4%) between our PAGE discovery stage studies with the published multi-ethnic meta-analysis [12].

### Interactions between CpG sites and behavioral factors on lipid levels

We first performed methylome-wide interaction analysis aiming to identify additional CpG sites whose associations with lipids were context specific, by allowing for interaction with smoking or alcohol intake status. We performed methylome-wide interaction analysis in the discovery studies (ARIC, WHI, and JHS, Supplemental Table 2). However, no CpG sites reached genome-wide significance (*P* < 1.18E-7).

We then performed stratified association analysis focusing on the novel CpG sites we identified in our PAGE analysis and those that had been previously associated in the literature. A total of 914, 316, and 16 top CpG sites from literature, the population-combined meta-analysis, and the population-specific meta-analysis, respectively, were tested for association with lipid levels in each smoking and alcohol intake stratum. In the smoking-stratified analysis, three and eight CpG sites associated with HDL-c and TG, respectively, showed significant differences across the three strata (*P* < 4.01E-5, Supplemental Fig. 1). For the three CpG sites displaying heterogeneous associations with HDL-c, all of them showed more significant association and larger effect sizes in nonsmokers compared to past or current smokers (Supplemental Fig. 1A-B). For the eight CpG sites showing heterogeneous associations with TG, cg16411857, which is previously reported for association with LDL-c, showed more significant association and larger effect sizes in current smokers (*P* = 1.02E-5) while the other seven CpG sites all showed more significant associations and larger effect sizes in nonsmokers (Supplemental Fig. 1B). These eight CpG sites included a newly identified CpG site, *SLC2A9*-cg02387843, which is associated with both HDL-c and TG levels in the population-combined meta-analysis and showed a more pronounced association with TG in the nonsmoking group (Supplemental Fig. 1B). In the alcohol-stratified analysis, one CpG site each in association with HDL-c (cg15804598) and TC (cg19377250), respectively, showed significant heterogeneity across strata (*P* < 4.01E-5, Supplemental Fig. 1). Both these two CpG sites were reported previously (cg15804598 for association with HDL-c and cg19377250 for association with TG) [12] and showed the largest effect sizes in moderate drinkers (Supplemental Fig. 1C and 1D).

### Pathway enrichment analysis

To better understand the pathways enriched for genes affected by lipid-associated CpG sites, we mapped all previously reported CpG sites as well as novel CpG sites identified in the current study to corresponding genes and then identified GO pathways that were over-represented by these genes. A total of 41, 57, 17, and 50 GO pathways are enriched for the mapped genes for HDL-c, LDL-c, TC, and TG, respectively (Supplemental Table 11, FDR adjusted *P* < 0.05). Among these significantly enriched pathways, 4 were shared across all four lipid traits, namely cholesterol biosynthetic process, cholesterol metabolic process, sterol biosynthetic process, and secondary alcohol biosynthetic process. Notably, the genes mapped to these four shared pathways were not the same across the four lipids traits (Supplemental Table 11), indicating distinct CpG sites and genes contributing to the enrichment of the same pathways across lipid traits. It is also worth mentioning that some enriched GO pathways with entirely different descriptions harbor the same set of genes. For example, the same five genes associated with LDL-c were mapped to both sterol metabolic process (GO:0016125) and secondary alcohol metabolic process (GO:1,902,652, supplemental Table 11). These connections across enriched pathways are displayed in Supplemental Fig. 2.

## Discussion

We report here a large-scale methylome-wide association analysis of blood lipids in diverse race and ethnicity populations and the first interaction analysis focusing on smoking and alcohol intake. We identified and replicated a total of 17 novel CpG sites in the meta-analysis. Although no additional novel CpG sites were identified in the interaction analysis, 13 CpG sites showed significant heterogeneity across smoking or alcohol intake groups in the stratified analysis. Pathway enrichment analysis, including both newly and previously identified CpG sites, revealed new inferences on lipid metabolism.

There are five population groups contributing to the combined meta-analysis, making it the most diverse study so far. Population-specific meta-analysis offered us an opportunity to identify potential population-specific signals and a CpG site in association with TG was revealed only in self-reported White participants. This CpG site, cg03584506, showed much larger effect sizes in White participants compared to African American participants (Table 2). It is also worth mentioning here that we included not only CpG sites measured on the Illumina Infinium HumanMethylation450 BeadChip but also ones on the Illumina Infinium MethylationEPIC BeadChip, which almost doubles the number of tested CpG sites. To maximize the sample size for the CpG sites measured on the Illumina Infinium MethylationEPIC BeadChip, we simply combined all available studies and summarized significant findings in Supplemental Table 6. Future studies are needed to validate these potential novel findings.

The overlap between genes mapped by lipid-associated CpG sites and genes identified in previous GWAS studies is limited. Among the 17 novel CpG sites identified in our analysis, 13 CpG sites mapped to a gene region while the others are located in gene desert areas. Only one of these 13 genes, *TNFAIP8* which is associated with TG in our study, has been reported in a published GWAS study for association with HDL-c and TG [38]. At the same time, 11 of the 13 mapped genes overlapped with reported genes from previous methylome-wide association studies for association with cardiometabolic traits or diseases, including type 2 diabetes [39], ischemic heart disease [39], body mass index [40–44], waist circumference [41, 42], and fasting insulin [45] (Supplemental Table 12). These observations demonstrate the strength of methylation studies for uncovering additional genes implicated in lipid metabolism and cardiometabolic traits and diseases. These 11 genes have also been reported for association with smoking and/or alcohol intake [46–49]. The mechanisms connecting these CpG sites to lipids as well as smoking and alcohol intake need further investigation. Although *C16orf38* and *FLJ43663* show no overlap with genes reported for association with cardiometabolic traits or diseases in methylome-wide association studies, there is strong association of *C16orf38* genetic variants with insulin-like growth factor 1 in previous GWAS studies [50, 51] and *FLJ43663* is linked to lipogenesis in hepatocytes [52].

Previous genome-wide genetic variant interaction analyses on lipids with smoking or alcohol intake identified additional genetic loci [13, 14], motivating us to implement the same strategy for CpG sites in the current study. However, no CpG site reached genome-wide significance in the interaction analysis. We further performed stratified analyses focusing only on newly identified CpG sites and previously reported ones and observed 11 and two CpG sites exhibiting significant heterogeneity across smoking and alcohol intake strata, respectively. The majority of the heterogeneous associations across smoking strata showed larger effect sizes in the nonsmoker group. The two heterogeneous associations across alcohol intake strata were both driven by larger effect sizes in the moderate drinker group, supporting previous studies which suggest an important influence of alcohol consumption on lipid levels. Follow-up analyses are needed to confirm these findings and the biological mechanisms underlying these observed heterogeneous associations require further investigation.

The pathways identified using CpG sites in the current study showed limited overlap with the ones identified using genetic variants from a previously published GWAS [53]. As expected, lipid metabolism-related pathways showed enrichment in both studies. In both studies, we observed that most of the enriched pathways were lipid-specific. It is worth noting that we identified immune response related pathways using LDL-associated CpG sites and it is well known that lipids are important factors in the host defense system [54]. We also identified blood cell differentiation related pathways using HDL-associated CpG sites, whose potential mechanisms have been explored in experimental studies in the setting of atherosclerosis progression [55]. Another possible explanation for this is the inadequate adjustment for estimated blood cell proportions in the association analyses.

There are three major limitations of our current study. First, examining additional factors, such as dietary habits, physical activity levels, geographic location, and prevalence of chronic diseases, might contribute to a better understanding of the association between CpG sites and lipid metabolism. These factors have been shown to influence lipid levels [56–58], and future studies are needed to explore the potential impact of these factors. Insufficient adjustment of confounding factors might also lead to the failure of replication of some CpG sites identified in the discovery stage. In addition, sex-stratified analyses could potentially help identify sex-specific associations that have been missed in the sex-combined analysis [59]. Secondly, whole blood-based DNA methylation was used in the current analysis while lipid metabolism-related tissues, namely liver, adipose tissue, and muscle, were not available for evaluation. Recent studies have explored DNA methylation patterns across tissues and the correlation varies based on genes, tissues, and the studied phenotypes [60–63]. Notably, findings from an epigenetic study on lipid-related metabolites indicated that differential methylation of multiple CpG sites persists in both whole blood and adipose tissue [64]. Another study found that differentially methylated CpG sites at well-established lipid-associated genes *ABCG1* and *SREBF1* were also associated with insulin resistance and BMI in blood, liver, and adipose tissues [65]. Whole blood has been the most commonly used biological material in epigenetic studies due to its easy accessibility and minimal invasiveness. Studies using DNA methylation measurements in whole blood benefit from much larger sample sizes compared to ones using tissue samples. Nevertheless, future studies on lipid metabolism in related tissues and experimental studies are needed to confirm findings from whole blood and to gain a more comprehensive picture of DNA methylation patterns across tissues. Thirdly, the cross-sectional design of our current study limits inference on temporality. Future Mendelian randomization analyses in large sample sizes are needed to clarify whether the CpG variations cause lipid level changes or are a consequence of lipid variability. There are two minor limitations of the current study. The limited sample sizes of Asian American and Native American populations prevented us from performing meaningful analyses. Participants in some of the included studies were originally selected for other phenotypes, which could potentially introduce a selection bias.

In conclusion, we identified 17 novel CpG sites in the methylome-wide association analysis and confirmed a considerable number of previously reported CpG sites. We identified 13 CpG sites which exhibit heterogeneous associations across smoking or alcohol intake strata. Our analyses provided additional information on the genes and pathways associated with lipid concentrations compared to GWAS studies.

## Supplementary Information

Supplementary material 1.

Supplementary material 2.

## Data Availability

Summary statistics of the methylome-wide association analyses and interaction analyses are available upon request.
