# Supplementary material for: Methylome-wide association analyses of lipids and modifying effects of behavioral factors in diverse race and ethnicity participants
Source: Clin Epigenetics. 2025 Apr 2;17:54. doi: 10.1186/s13148-025-01859-3 (PMC11967142; doi:10.1186/s13148-025-01859-3)
Supplement: Supplementary file 1 — Supplementary material 1. [file 13148_2025_1859_MOESM1_ESM.docx]

**Supplemental Figures**

**Fig S1. CpG sites showing significant heterogeneity across smoking or alcohol intake strata. (A) association with HDL-c across smoking strata; (B) association with TG across smoking strata; (C) association with HDL-c across alcohol intake strata; (D) association with TC across alcohol intake strata.**

**(A)**

**
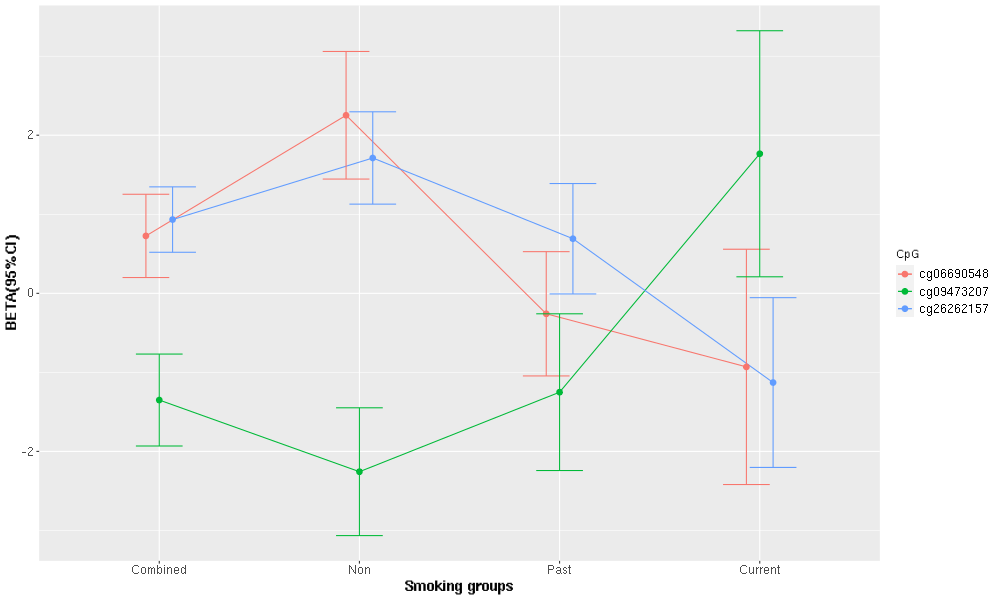
**

**(B)**

**
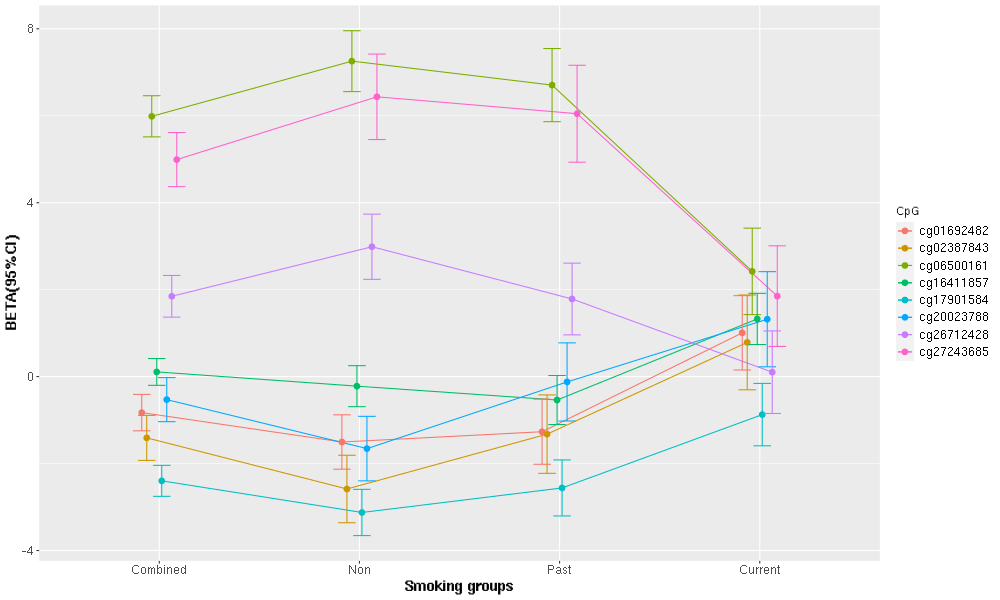
**

**(C)**

**
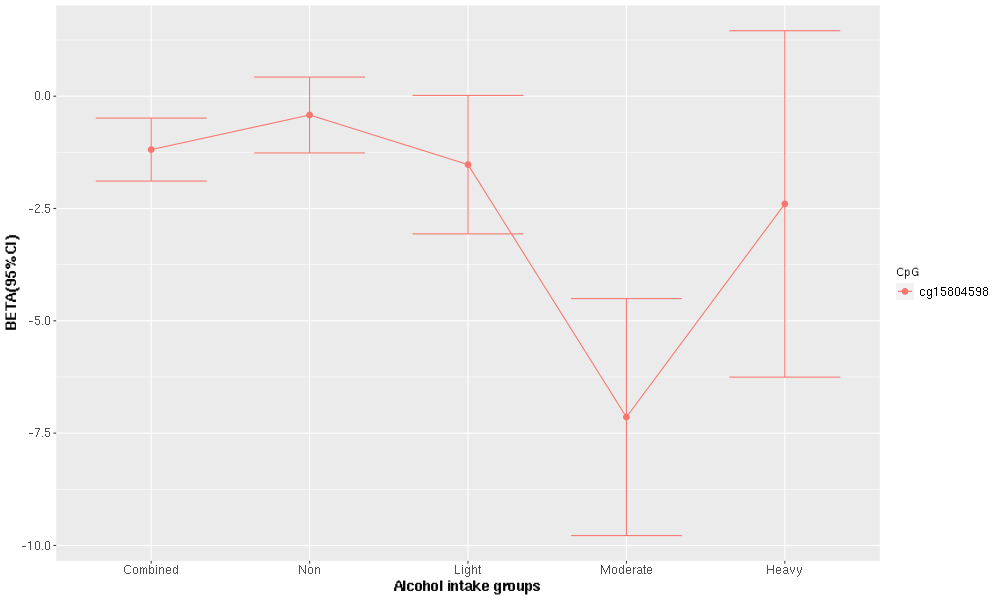
**

**(D)**

**
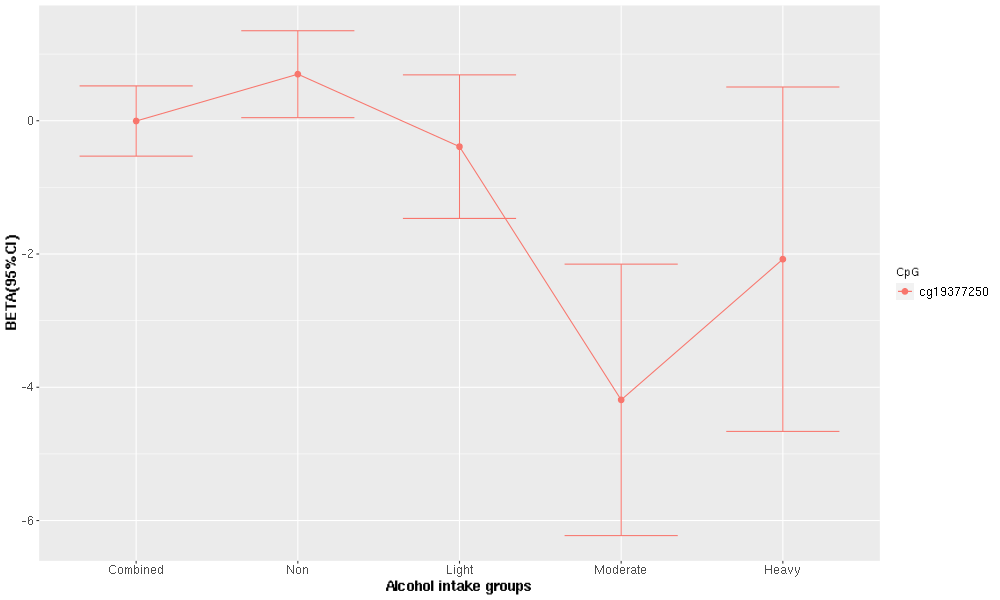
**

**Fig S2. GO enrichment maps. (A) HDL-c; (B) LDL-c; (C) TC; (D) TG. Each dot represents a pathway. The size of the dot represents the number of genes in the pathway and the color of the dot represents the P value for enrichment. Dots connected by gray lines indicate that these pathways harbor shared genes.**

**(A)**


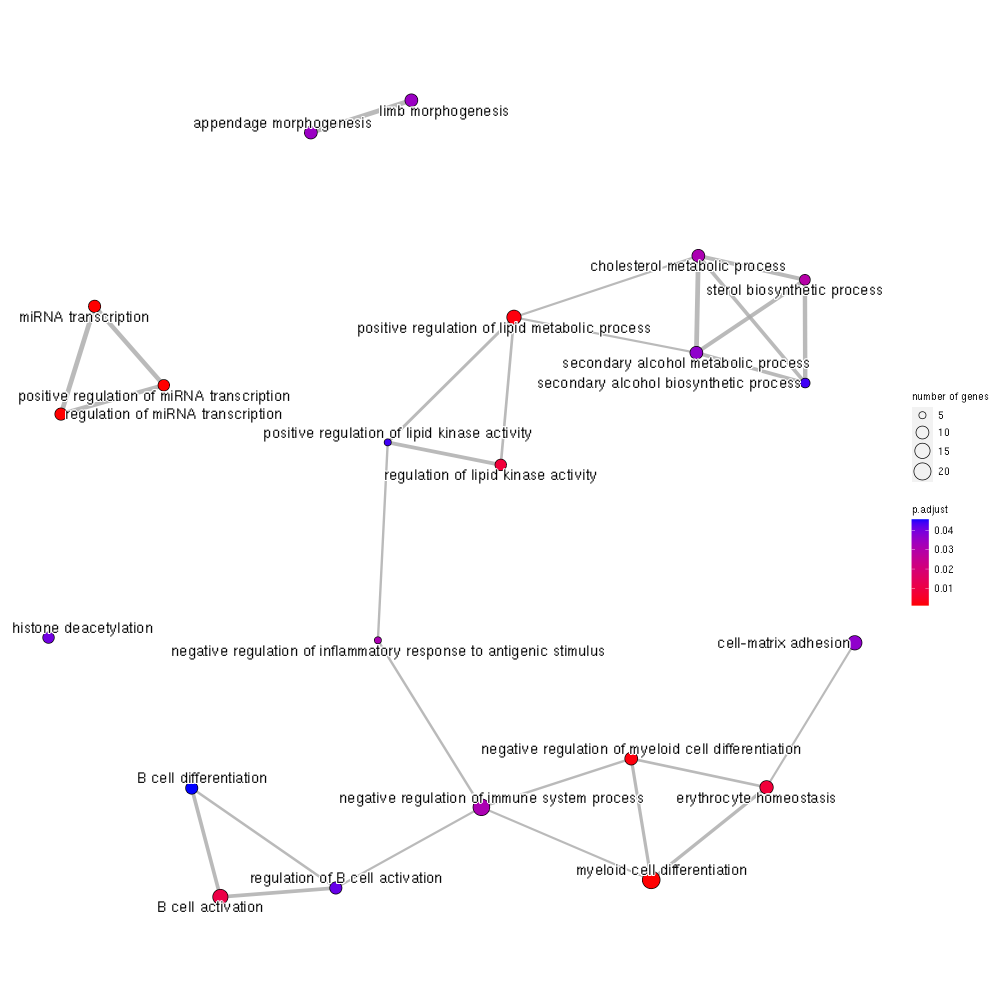


**(B)**


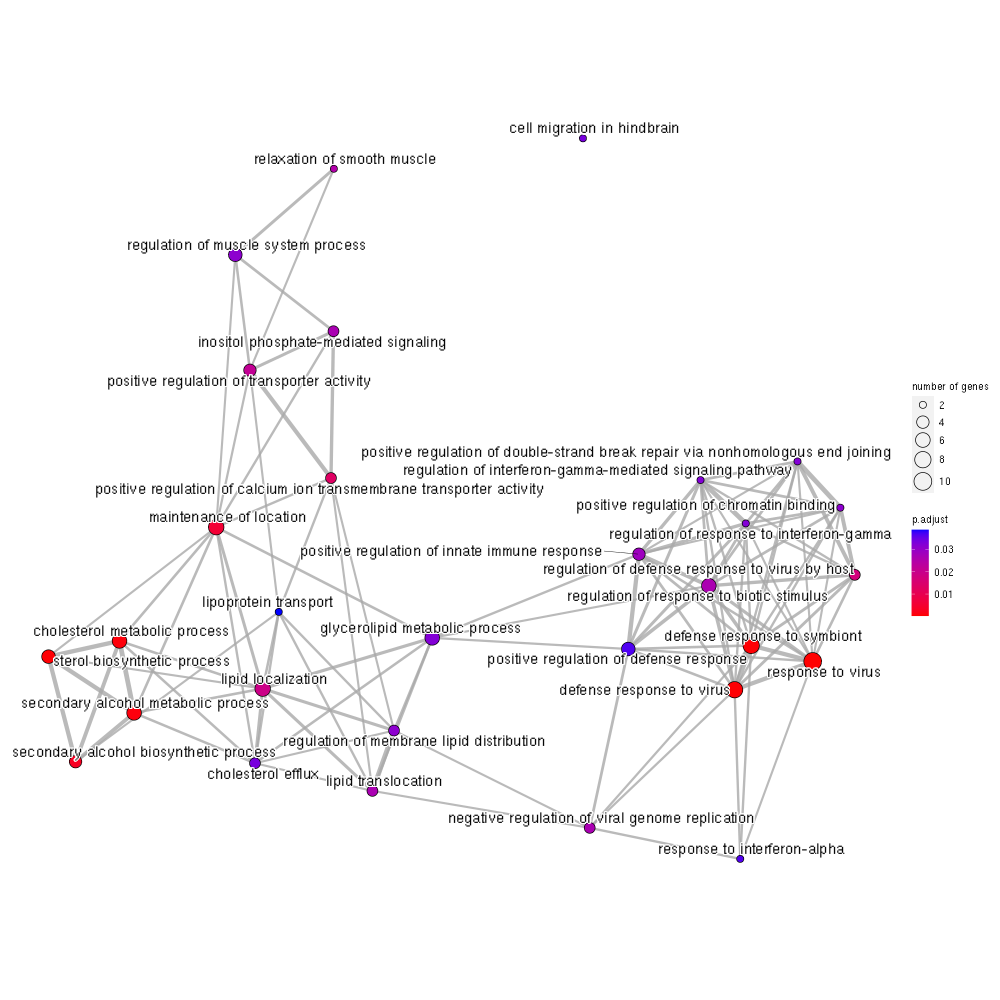


**(C)**


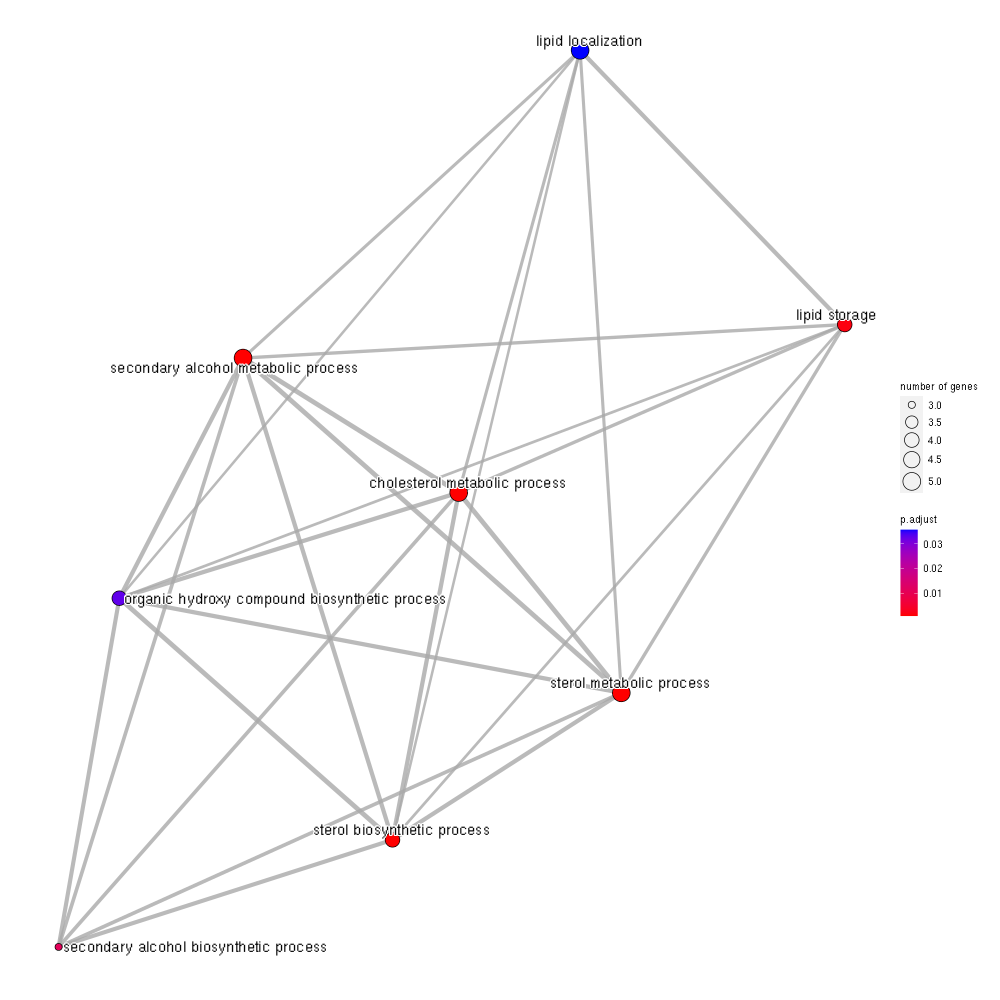


**(D)**


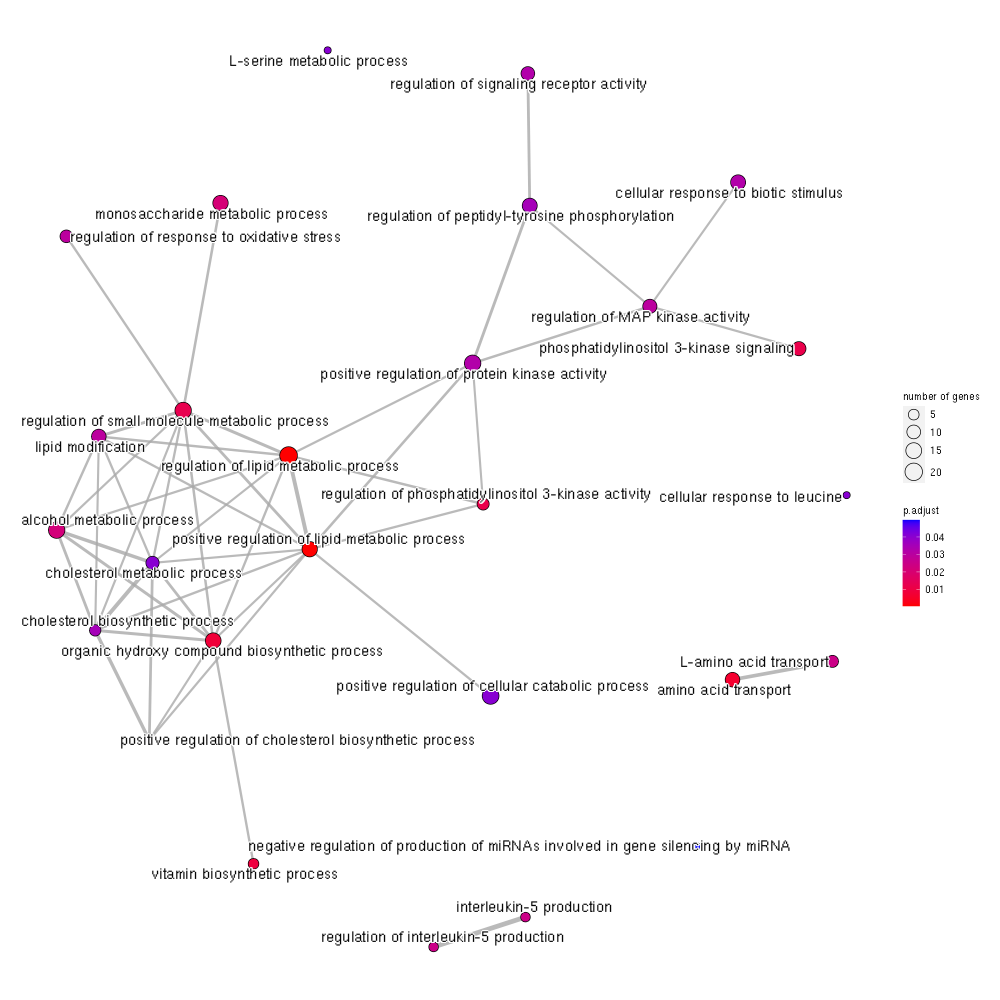


**Supplemental Materials**

**Participating studies**

Amish

White blood cell (WBCs) samples were obtained from relatively healthy participants of several studies including the Heredity and Phenotype Intervention (HAPI)^1^, the Pharmacogenomics of Antiplatelet Intervention (PAPI)^2^, and the Amish Wellness Study (WELLNESS, initiated in 2010 to provide wellness screening for cardiometabolic health). Individuals 20 years of age or older were recruited from the Amish community of Lancaster County, PA between 2003 and 2015. The Old Order Amish founder population was chosen because their genetic homogeneity might allow for easier discernment of genetic effects on complex phenotypes.

Probes were removed if they did not pass QC detection P values >0.01. In order to avoid false signals due to underlying polymorphisms, probes were removed if a SNP with MAF>0.05 in Europeans was present within 50 base pairs of the CpG site. We also removed a list of probes identified as cross-hybridizing to regions of the genome outside of their intended target. Chen et al used the 1000 Genome database to identify probes with a genetic polymorphism and cross-reactive probes^3^. Following probe QC procedure, we performed preprocessing of raw microarray intensity data using minfi. We used the quantile normalization procedure that has been previously described^4^. Cell type composition variation across samples was estimated using minfi software and a reference panel^5^. Samples with swapped gender labels were removed.

Raw lipid phenotype values were obtained by HAPI, PAPI, and Wellness study protocols. Adjustments to raw lipid levels were made for participants taking lipid-lowering drugs. MMAP software (https://mmap.github.io/) was used to obtain inverse normal trait values without controlling for other covariates.

Methylome-wide association analyses were run using in-house MMAP software (<https://mmap.github.io/>) and controlling for white blood cell proportions, age, gender, plate, and, when applicable to the trait, we included high effect size SNPs enriched in the Amish population and previously studied by our group. These two variants are the *APOC3* p.R19X variant, associated with lower triglyceride levels^6^, and the *APOB* p.R3527Q, associated with increased LDL-c levels^7^.

ARIC

The Atherosclerosis Risk in Communities (ARIC) study included data from two ancillary studies of African American and White populations^8,9^. ARIC is an ongoing prospective cohort study investigating the etiology of CHD in four US communities: Forsyth County, NC; Jackson, MS; suburbs of Minneapolis, MN; Washington County; MD. Participants were aged 45–64 at baseline and followed up in each community over 30 years with 10 study visits (visit 11 is in progress). DNA methylation was measured in 2879 African American and 1100 White participants from ARIC in visit 2 (1990–1992) or visit 3 (1993–1995). For the subjects that overlap between ARIC and JHS, all JHS participants were retained, after restrictions based on relatedness. Genomic DNA was extracted from peripheral whole blood samples and bisulphite and converted with standardized procedures. The HumanMethylation450 microarray (Illumina Inc.; San Diego, CA, USA) was used to measure DNA methylation at up to 485,577 CpG sites by analyzing sequencing-based genotyping. Methylation scores for each CpG site as a beta (β) value that ranged from 0 to 1 (non-methylated to completely methylated) were calculated, according to the intensity ratio of detected methylation, after adjustments for potential batch effects, white blood cell counts/estimates, non-normality, and design probe differences using BMIQ. Quality control procedures include filtering of known cross-reactive and polymorphic probes, and by call rate. Lipid measurements, medication use, smoking and alcohol intake were collected at both visit 2 and 3 and were included for analyses.

CHS

The CHS is a population-based cohort study of risk factors for coronary heart disease and stroke in adults ≥65 years conducted across four field centers^10^. The original predominantly White participants cohort of 5,201 persons was recruited in 1989-1990 from random samples of the Medicare eligibility lists; subsequently, an additional predominantly African American cohort of 687 persons was enrolled for a total sample of 5,888. CHS was approved by institutional review committees at each field center and individuals in the present analysis had available DNA and gave informed consent including consent to use of genetic information for the study of cardiovascular disease.

DNA methylation was measured on a randomly selected subset of 336 White participants and 329 African American participants who participated in the 3rd annual follow-up visit (study year 5) and had DNA available from that visit. The White participants had no baseline history of coronary vascular disease (defined as coronary heart disease, congestive heart failure, peripheral vascular disease, valvular heart disease, stroke, or transient ischemic attack). An additional 105 participants were selected based on drug use and availability of DNA at both the 3rd and 7th annual follow-up visits (study years 5 and 9).

Methylation measurements were performed at the Institute for Translational Genomics and Population Sciences at the Harbor-UCLA Medical Center Institute for Translational Genomics and Population Sciences (Los Angeles, CA). DNA was extracted from Buffy coat fractions and subsequently underwent bisulfite conversion using the EZ DNA Methylation kit (Zymo Research, Irvine, CA). Methylation was then assayed using the Infinium HumanMethylation450 BeadChip (Illumina Inc, San Diego, CA).

Quality control was performed in the minfi R package (version 1.12.0, http://www.bioconductor.org/packages/release/bioc/html/minfi.html)^5^. Samples with low median intensities of below 10.5 (log2) across the methylated and unmethylated channels, samples with a proportion of probes falling detection of greater than 0.5%, samples with QC probes falling greater than 3 standard deviations from the mean, sex-check mismatches, failed concordance with prior genotyping or > 0.5% of probes with a detection p-value > 0.01 were removed. Probes with >1% of values below detection were removed. In total, 11 samples were removed for sample QC resulting in a sample of 323 White participants and 326 African American participants. Methylation values were normalized using the SWAN quantile normalization method^11^. Since white blood cell proportions were not directly measured in CHS they were estimated from the methylation data using the Houseman method^12^.

GENOA

The Genetic Epidemiology Network of Arteriopathy (GENOA) study is a community-based study of hypertensive sibships that was designed to investigate the genetics of hypertension and target organ damage in African Americans from Jackson, Mississippi and non-Hispanic whites from Rochester, Minnesota (Daniels, 2004). In the initial phase of GENOA (Phase I: 1996-2001). All members of sibships containing ≥ 2 individuals with essential hypertension clinically diagnosed before age 60 were invited to participate, including both hypertensive and normotensive siblings. Exclusion criteria of the GENOA study were secondary hypertension, alcoholism or drug abuse, pregnancy, insulin-dependent diabetes mellitus, or active malignancy. Eighty percent of African Americans (1,482 subjects) and 75% of non-Hispanic whites (1,213 subjects) from the initial study population returned for the second examination (Phase II: 2001-2005). Study visits were made in the morning after an overnight fast of at least eight hours. Demographic information, medical history, clinical characteristics, lifestyle factors, and blood samples were collected in each phase. Written informed consent was obtained from all subjects and approval was granted by participating institutional review boards. DNA methylation levels were measured only in African American participants.

A total of 1106 samples at Phase I and 304 samples at Phase II were assessed using the Illumina HumanMethylationEPIC BeadChip. First, raw IDAT files were imported using Minfi R package^5^. We used the shinyMethyl R package^13^ to visualize the raw intensity data and identify sex mismatches and outliers, which were removed. We also obtained detection p-value for each sample at each probe, and individual probes with detection p-value <1E^-16^ were considered to be detected successfully^14^. Samples and probes with detection rate <10% were removed. Samples with incomplete bisulfite conversion identified using the QCinfo() function in the ENmix R package were removed^15^. We also checked sample identity using the 59 SNP probes implemented in the EPIC chip and removed mismatched samples. Next, Noob was used for individual background and dye-bias normalization^13^. Since two types of probes are present on the EPIC BeadChip (Infinium I and Infinium II), we used the Regression on Correlated Probes (RCP) method to adjust for probe-type bias^16^. After exclusions, a total of 857,121 probes in 1,100 samples at Phase I and 294 samples at Phase II were available for analysis.

All lipid measures and covariates were taken from GENOA Phase I data. Total cholesterol (TC), HDL, and triglycerides (TG) were measured from blood samples, and LDL was calculated as TC-HDL-(TG/5). Raw values were then adjusted according to the drug intake constants in the analysis protocol, and TG was natural log transformed. Participants with TG>400 were removed, and outliers greater than 5 standard deviations from the mean were set to missing. Smoking was categorized into never, former, and current. Alcohol intake was categorized into none (0 drinks per week), light (0<drinks<=3), moderate (3<drinks<=14 for males and 3<drinks<=7 for females), and heavy (drinks>14 for males and drinks>7 for females). Summary statistics are shown below. After exclusions, there was a total N=942 with complete data on all covariates and TC or TG, and N=939 with complete data on all covariates and HDL or LDL.

A two-stage regression model was used to assess relationships between lipids and methylation beta values. First, each lipid measure was regressed on age and gender. The residuals from this model were then inverse-normalized and used as the outcomes in the methylome-wide association analysis. Part 1 controlled for white blood cell proportions (CD8T, CD4T, NK, BCELL, MONO, and GRAN values estimated using the Houseman method), the first ten principal components (PCs), row, column, and plate (random effect). Due to the family structure in GENOA, a random effect for family ID was also included. The lmer function within R packages lme4 and lmerTest was used for this purpose. For the genome-wide interaction analysis, smoking/alcohol intake and its interaction with the CpG beta value were also included.

JHS

The Jackson Heart Study (JHS) was initiated in 1998 as a longitudinal investigation of genetic and environmental risk factors associated with the disproportionate burden of cardiovascular disease in African Americans. This study is an extension of the ARIC study, with additional recruitment of AA men and women, aged 35-84, in Jackson, Mississippi, as well as for the family study component only adults 21 and older. JHS participants received three back-to-back clinical examinations (Exam 1, 2000-2004; Exam 2, 2005-2008; and Exam 3, 2009-2013) that have generated extensive longitudinal data on traditional and putative cardiovascular disease risk factors. A fourth exam is ongoing. DNA methylation and lipid measurements used for this study were all collected at baseline and retained one individual per family group (comparing to parents and siblings, and overlap participants with ARIC were excluded from JHS). JHS methylation data has been previously described^17^. The JHS data for this study was obtained with a Data and Materials Distribution Agreement for JHS ASC# P1231.

MEC

The MEC is a prospective study of > 215,000 men and women of five racial and ethnic groups: Black or African American, Asian or Asian American (Japanese), Pacific Islander (Native Hawaiian), Hispanic/Latina/o, and White participants, recruited from the state of Hawaii and from Southern California, primarily Los Angeles County, between 1993 and 1996^16^. The MEC has a biorepository of blood specimens from approximately 70,000 participants, who provided samples around 10 years after cohort entry. For the present study, we focused our analysis on a subcohort of participants who had genomic ancestry, DNA methylation and CRP measured and were current smokers with no lung cancer history at the time of biospecimen collection^17^. For genomic ancestry and DNA methylation, individuals were selected as part of a subcohort of current smokers at time of blood draw who were genotyped using the Illumina Human1M-Duo BeadChip (1,199,187 single nucleotide polymorphisms [SNPs])^20^ and had DNA methylation measured by Illumina MethEPIC chip. High sensitivity CRP (hsCRP) was measured from fasting blood samples obtained between 1994 and 2016^21^.

MESA

The Multi-Ethnic Study of Atherosclerosis (MESA) is a study of the characteristics of subclinical cardiovascular disease (disease detected non-invasively before it has produced clinical signs and symptoms) and the risk factors that predict progression to clinically overt cardiovascular disease or progression of the subclinical disease^22^. MESA researchers study a diverse, population-based sample of 6,814 asymptomatic men and women aged 45-84. Thirty-eight percent of the recruited participants are white, 28 percent African-American, 22 percent Hispanic, and 12 percent Asian, predominantly of Chinese descent. Participants were recruited from six field centers across the United States: Wake Forest University, Columbia University, Johns Hopkins University, University of Minnesota, Northwestern University and University of California - Los Angeles. Each participant received an extensive exam and determination of coronary calcification, ventricular mass and function, flow-mediated endothelial vasodilation, carotid intimal-medial wall thickness and presence of echogenic lucencies in the carotid artery, lower extremity vascular insufficiency, arterial waveforms, electrocardiographic (ECG) measures, standard coronary risk factors, sociodemographic factors, lifestyle factors, and psychosocial factors. Selected repetition of subclinical disease measures and risk factors at follow-up visits allows study of the progression of disease. Blood samples have been assayed for putative biochemical risk factors and stored for case-control studies. DNA has been extracted and lymphocytes cryopreserved (for possible immortalization) for study of candidate genes and possibly, genome-wide scanning, expression, and other genetic techniques. Participants are being followed for identification and characterization of cardiovascular disease events, including acute myocardial infarction and other forms of coronary heart disease (CHD), stroke, and congestive heart failure; for cardiovascular disease interventions; and for mortality.

Lipid measurements from exam 1 (baseline) were used, adjusted for medication, and transformed as specified in the Methods section. DNA methylation was measured by Illumina’s Methylation EPIC BeadChip (850 K) at exam 1. Quality control (QC) on DNA methylation (DNAm) data was applied and methylation positions with low methylation variation (i.e., the standard deviation of methylation Beta-values < 0.02) were filtered out. The epigenetic analyses were then performed on the filtered methylation positions. Details of DNA methylation profiling have been described previously ^23^.

WHI

WHI is a long-term, prospective, multi-center cohort study investigating post-menopausal women’s health in the US^24^. The WHI data for this paper comes from four ancillary studies [EMPC (AS315), BA23, AS311, and the Long Life Study (LLS)]. The Epigenetic Mechanisms of PM-Mediated CVD Risk (WHI-EMPC) study assessed epigenetic mechanisms underlying associations between ambient particulate matter air pollution and cardiovascular disease within the WHI Clinical Trials (CT). From this population, DNA methylation was measured in 2200 randomly selected participants (Screening Visit,) (CT, n = 2200), and remeasured in 200 participants at a second visit (stage 2: Annual Visit (AV) 3, or AV6). For this study participants were selected from the screening visit. If that data was not available for both DNA methylation and lipid measurements, data from a follow-up visit was used. The Broad Agency Announcement 23 (WHI-BAA23) study, also known as Integrative Genomics and Risk of CHD and Related Phenotypes in the Women's Health Initiative, was a case–control study assessing predictors of coronary heart disease (CHD) within the WHI CT (n = 1664) and observational study (OS, n = 442), where cases were identified using eight biomarkers of CHD. By design, WHI-BAA23 oversampled African Americans and Hispanic/Latino Americans and required all participants to have undergone genome-wide genotyping and profiling of seven cardiovascular disease biomarkers. Ancillary Study 311 (AS311) was a matched case–control study of bladder cancer among women within the WHI CT (n = 405) and OS (n = 455). The LLS participants were CT and OS participants with follow-up visits ranging from 14 to 19 years after initial enrollment (hence the higher average age relative to the other studies); participants do not overlap with those in the studies mentioned above. While the specimen analyzed for AS311, AS315, and BAA23 were from visits at (or close to) baseline, the specimen analyzed for the LLS study were obtained about 20 years later in 2012/13.

**References**

1. Mitchell, B. D. *et al.* The genetic response to short-term interventions affecting cardiovascular function: Rationale and design of the Heredity and Phenotype Intervention (HAPI) Heart Study. *Am Heart J* **155**, (2008).

2. Shuldiner, A. R. *et al.* Association of cytochrome P450 2C19 genotype with the antiplatelet effect and clinical efficacy of clopidogrel therapy. *JAMA* **302**, (2009).

3. Chen, Y. A. *et al.* Discovery of cross-reactive probes and polymorphic CpGs in the Illumina Infinium HumanMethylation450 microarray. *Epigenetics* **8**, (2013).

4. Wu, Z. & Aryee, M. J. Subset quantile normalization using negative control features. *Journal of Computational Biology* **17**, (2010).

5. Aryee, M. J. *et al.* Minfi: A flexible and comprehensive Bioconductor package for the analysis of Infinium DNA methylation microarrays. *Bioinformatics* **30**, (2014).

6. Pollin, T. I. *et al.* A null mutation in human APOC3 confers a favorable plasma lipid profile and apparent cardioprotection. *Science (1979)* **322**, (2008).

7. Shen, H. *et al.* Familial defective apolipoprotein B-100 and increased low-density lipoprotein cholesterol and coronary artery calcification in the old order amish. *Arch Intern Med* **170**, (2010).

8. The ARIC Investigators. The Atherosclerosis Risk In Communities (ARIC) Study: Design and Objectives. *Am J Epidemiol* **129**, (1989).

9. Anderson, G. *et al.* Design of the Women’s Health Initiative clinical trial and observational study. *Control Clin Trials* **19**, (1998).

10. Fried, L. P. *et al.* The cardiovascular health study: Design and rationale. *Ann Epidemiol* **1**, (1991).

11. Maksimovic, J., Gordon, L. & Oshlack, A. SWAN: Subset-quantile within array normalization for illumina infinium HumanMethylation450 BeadChips. *Genome Biol* **13**, (2012).

12. Houseman, E. A. *et al.* DNA methylation arrays as surrogate measures of cell mixture distribution. *BMC Bioinformatics* **13**, (2012).

13. Fortin, J. P., Fertig, E. & Hansen, K. shinyMethyl: Interactive quality control of Illumina 450k DNA methylation arrays in R. *F1000Res* **3**, (2014).

14. Lehne, B. *et al.* A coherent approach for analysis of the Illumina HumanMethylation450 BeadChip improves data quality and performance in epigenome-wide association studies. *Genome Biol* **16**, (2015).

15. Xu, Z., Niu, L., Li, L. & Taylor, J. A. ENmix: A novel background correction method for Illumina HumanMethylation450 BeadChip. *Nucleic Acids Res* **44**, (2016).

16. Niu, L., Xu, Z. & Taylor, J. A. RCP: A novel probe design bias correction method for Illumina Methylation BeadChip. in *Bioinformatics* vol. 32 (2016).

17. Raffield, L. M. *et al.* Coagulation factor VIII: Relationship to cardiovascular disease risk and whole genome sequence and epigenome-wide analysis in African Americans. *Journal of Thrombosis and Haemostasis* **18**, (2020).

18. Kolonel, L. N. *et al.* A multiethnic cohort in Hawaii and Los Angeles: Baseline characteristics. *Am J Epidemiol* **151**, (2000).

19. Murphy, S. E. *et al.* Nicotine N-glucuronidation relative to N-oxidation and C-oxidation and UGT2B10 genotype in five ethnic/racial groups. *Carcinogenesis* **35**, (2014).

20. Patel, Y. M. *et al.* The contribution of common genetic variation to nicotine and cotinine glucuronidation in multiple ethnic/racial populations. *Cancer Epidemiology Biomarkers and Prevention* **24**, (2015).

21. Morimoto, Y. *et al.* Ethnic differences in serum adipokine and C-reactive protein levels: The multiethnic cohort. *Int J Obes* **38**, (2014).

22. Bild, D. E. *et al.* Multi-Ethnic Study of Atherosclerosis: Objectives and design. *Am J Epidemiol* **156**, (2002).

23. Hu, X. *et al*. Multi-ancestry epigenome-wide analyses identify methylated sites associated with aortic augmentation index in TOPMed MESA. *Sci Rep* **13**, (2023).

24. Anderson, G. *et al.* Design of the Women’s Health Initiative clinical trial and observational study. *Control Clin Trials* **19**, (1998).
